# The Impact of the COVID-19 Pandemic on Drug Use Behaviors, Fentanyl Exposure, and Harm Reduction Service Support among People Who Use Drugs in Rural Settings

**DOI:** 10.3390/ijerph19042230

**Published:** 2022-02-16

**Authors:** Rebecca S. Bolinski, Suzan Walters, Elizabeth Salisbury-Afshar, Lawrence J. Ouellet, Wiley D. Jenkins, Ellen Almirol, Brent Van Ham, Scott Fletcher, Christian Johnson, John A. Schneider, Danielle Ompad, Mai T. Pho

**Affiliations:** 1 Department of Sociology, Southern Illinois University, 475 Clocktower Drive, Room 323A, Carbondale, IL 62901, USA; 2 Center for Drug Use and HIV|HCV Research, School of Global Public Health, New York University, New York, NY 10003, USA; suzanmwalters@gmail.com (S.W.); danielle.ompad@nyu.edu (D.O.); 3 Department of Family Medicine and Community Health, Madison School of Medicine and Public Health, University of Wisconsin, Madison, WI 53792, USA; elizabeth.salisbury-afshar@fammed.wisc.edu; 4 Division of Epidemiology and Biostatistics/COIP (MC 923), School of Public Health, University of Illinois Chicago, 1603 W. Taylor, Chicago, IL 60607, USA; ljo@uic.edu; 5 Epidemiology and Biostatistics, Department of Population Science and Policy, Southern Illinois University School of Medicine, Springfield, IL 62794, USA; wjenkins@siumed.edu; 6 Chicago Center for HIV Elimination, University of Chicago, Chicago, IL 60637, USA; ealmirol@medicine.bsd.uchicago.edu; 7 Center for Rural Health and Social Service Development, Southern Illinois University School of Medicine, Carbondale, IL 62901, USA; bvanham49@siumed.edu; 8 The Community Action Place, Inc., 1400 N. Wood Road Suite 7, Murphysboro, IL 62966, USA; scott.fletcher@tcapinc.org; 9 Department of Medicine, Section of Infectious Diseases and Global Health, University of Chicago, Chicago, IL 60637, USA; cjohnson5@medicine.bsd.uchicago.edu (C.J.); jschnei1@medicine.bsd.uchicago.edu (J.A.S.); mpho1@uchicago.edu (M.T.P.); 10 Department of Epidemiology, School of Global Public Health, New York University, New York, NY 10003, USA

**Keywords:** fentanyl beans, fentanyl buttons, methamphetamine, COVID-19, rural, PWID, harm reduction

## Abstract

Background: The COVID-19 pandemic has worsened the opioid overdose crisis in the US. Rural communities have been disproportionately affected by opioid use and people who use drugs in these settings may be acutely vulnerable to pandemic-related disruptions due to high rates of poverty, social isolation, and pervasive resource limitations. Methods: We performed a mixed-methods study to assess the impact of the pandemic in a convenience sample of people who use drugs in rural Illinois. We conducted 50 surveys capturing demographics, drug availability, drug use, sharing practices, and mental health symptoms. In total, 19 qualitative interviews were performed to further explore COVID-19 knowledge, impact on personal and community life, drug acquisition and use, overdose, and protective substance use adaptations. Results: Drug use increased during the pandemic, including the use of fentanyl products such as gel encapsulated “beans” and “buttons”. Disruptions in supply, including the decreased availability of heroin, increased methamphetamine costs and a concomitant rise in local methamphetamine production, and possible fentanyl contamination of methamphetamine was reported. Participants reported increased drug use alone, experience and/or witness of overdose, depression, anxiety, and loneliness. Consistent access to harm reduction services, including naloxone and fentanyl test strips, was highlighted as a source of hope and community resiliency. Conclusions: The COVID-19 pandemic period was characterized by changing drug availability, increased overdose risk, and other drug-related harms faced by people who use drugs in rural areas. Our findings emphasize the importance of ensuring access to harm reduction services, including overdose prevention and drug checking for this vulnerable population.

## 1. Introduction

The COVID-19 pandemic has had wide-ranging, global impacts on health outcomes for communities of high vulnerability, including people who use drugs (PWUDs). Deaths from drug overdoses, which were previously stable between 2017 to September 2019, began rising in nearly every state in the months before the pandemic and surged in April 2020 (provisional data from the Centers for Disease Control and Prevention) [1]. Overdose deaths continued to rise, reaching 75,673 deaths by April 2021 (provisional data from the Centers for Disease Control and Prevention) [2]. In Illinois, despite legislative action and initiatives expanding naloxone access, including the enrollment of over 130 drug overdose programs supported by state and federal funding, overdose deaths increased by 32.6% between September 2019 and September 2020 [3,4,5]. This trend reversed progress that had been made in the year prior. Changes in drug supply chains during the pandemic, particularly the greater availability of synthetic opioids such as fentanyl, have been postulated as driving factors for this increase [6,7,8]. According to the US Drug Enforcement Agency, record numbers of fentanyl seizures have occurred across the country where fentanyl is trafficked along well-established supply routes built up from prior heroin, cocaine, and cannabis trades [9,10,11].

In recent years, rural communities have been disproportionately affected by the opioid and methamphetamine epidemics [12,13]. These communities are at increased risk due to socioeconomic vulnerability and limited substance use disorder treatment, mental health treatment, and harm reduction services, including syringe service programs and naloxone distribution. PWUDs in rural areas face multiple barriers to good health, including chronic homelessness, food insecurity, and stigmatizing experiences in health care and the community [14,15]. During the first three quarters of 2020, opioid-related overdose mortality increased by 39% in rural Illinois counties as compared to 11% in small urban and suburban counties [16]. COVID-19 has likely exacerbated these vulnerabilities, particularly where limited public health resources previously supporting overdose surveillance and prevention have been diverted to address the pandemic. Little direct information is available on the impact of the pandemic and the associated social mitigation measures on drug availability, how people acquire and use drugs, and access to essential prevention and treatment services in rural settings.

Within the context of an ongoing clinical trial of harm reduction service expansion for PWUDs in southern Illinois, we performed an exploratory, mixed-methods study to assess changes to drug access, drug use behavior, and harm reduction services since the onset of the COVID-19 pandemic. We adapted an existing survey and performed qualitative interviews to better understand the contextual factors and social stressors influencing perceptions of risk and safety related to injection drug use, as well as adaptations in behavior. 

## 2. Materials and Methods

The Ending Transmission of HIV, Hepatitis C, Sexually Transmitted Diseases and Overdose in Rural Communities of People Who Inject Drugs (ETHIC) clinical trial is a cooperative agreement evaluating the epidemiology of the opioid epidemic, related infectious diseases, and barriers to the expansion of harm reduction services in rural settings. Our research was conducted in the Illinois counties of the Delta Regional Authority (DRA) [17], a federal-state partnership for economic development spanning 252 counties and parishes in eight states along the Mississippi River and southern Alabama. These southern Illinois counties are mainly rural and dominated by agricultural industries, with 45% of residents living below 200% of the poverty level [17,18]. All counties are designated as shortage areas of primary and mental health care [19,20].

ETHIC eligibility requirements include age ≥15 years, residence in the counties described above, English speaking, and reported use of opioids for nonmedical purposes or injecting any type of drug for nonmedical reasons within the 30 days prior to the interview. Participants were recruited using respondent-driven sampling, an incentivized snowball type recruitment strategy that is effective in reaching vulnerable populations, including persons who use drugs [21]. Index participants, or “seeds”, were recruited from the client base of a grassroots, peer-led harm reduction organization serving the geographic area of the study setting. Seeds were given a unique code word and asked to recruit up to six peers who were eligible and may be interested in participating in the study. Their recruits were then asked to recruit peers through the same process. Recruiters were incentivized for each person they referred who successfully enrolled in the study. Participants completed a baseline survey assessment that captured demographics, social determinants of health, mental health screening, drug use behavior, sexual risk behavior, access to harm reduction services, and substance use treatment and medical care utilization. Participants who were not active clients of the harm reduction organization were offered a referral, including an immediate “warm handoff” to service delivery after baseline survey completion.

ETHIC enrollment for the baseline survey began in June of 2018 until July 2019 and was paused until August 2020 due to a planned study transition, followed by delays resulting from COVID-19 restrictions. During this delay, COVID-related survey questions were developed to assess the impact of the pandemic on social supports, drug availability, drug use behaviors, overdose experience, mental health, and access to harm reduction services. A convenience sample of ETHIC participants completed the COVID-19 related survey items and/or the qualitative interview. 

We reported descriptive statistics of the baseline assessment and COVID-19 survey responses using STATA (Software Version 16, StataCorp LLC, College Station, TX, USA). For participants who completed the baseline assessment during the pre-pandemic phase of data collection (i.e., before August 2020), there was a time gap between the baseline and COVID-19 survey administration that ranged from 15 to 25 months. For participants who were enrolled after July 2020, the gap between the baseline and COVID-19 survey questions ranged from 0 to 6 months. Due to potential differences between participants who were enrolled pre- and during pandemic periods, we compared the baseline characteristics of these two groups using a nonparametric Fisher exact test.

A semistructured, qualitative interview guide was developed to explore more deeply study participants’ COVID-19 knowledge, impact on personal and community life, drug acquisition and use, overdose, and experience with harm reduction. A subset of those who completed the initial baseline assessment was asked to complete the qualitative interviews. Interviews were conducted over the phone, recorded, transcribed, and coded by authors R.B. and S.W. using the existing codebook developed from the previous projects for all domains except COVID-19. New codes for COVID-19 were developed by using line-by-line coding and then grouping thematically [22]. Qualitative data were processed and analyzed using Dedoose (Version 8.3.17, SocioCultural Research Consultants LLC, Los Angeles, CA, USA). 

Participants were compensated for participating in the surveys, qualitative interviews, and peer recruitment. Study activities were approved by the University of Chicago and New York University Institutional Review Boards.

## 3. Results

### 3.1. Participant Characteristics 

A total of 50 participants completed the COVID-19 survey, and 19 individuals participated in the qualitative interview, of which 14 participants completed both the COVID survey and qualitative interview. Demographic characteristics, social determinants of health, mental health symptoms, drug use behaviors, and past overdose events were drawn from the ETHIC baseline survey (*n* = 50). Of those completing the baseline survey, 18 participants provided baseline data during the pre-COVID period and 32 after the onset of the pandemic (Table A1). The mean age of respondents was 40 years, 48% identified as female, and the majority were white (88%) (Table 1). In terms of education, 42% had some high school, and 32% had graduated high school or equivalent. The sample had high rates of homelessness (62%) and accessing food supports in the past six months (92%). The most common drugs used in the 30 days prior to completion of the baseline survey per self-report were methamphetamines (96%), heroin (60.5%), and fentanyl (45.2%). A comparison of baseline characteristics between participants who were enrolled pre- and post-pandemic was performed (Table A2). There were no significant differences in demographic characteristics, social determinants (e.g., income, homelessness, accessing food support), injection drug use, or use of methamphetamine, heroin, or fentanyl. However, participants reported more recent drug use in the pre-pandemic period, compared with the pandemic period for prescription anxiety drugs such as “Xanax, Valium, and Klonopin” (50% pre-pandemic vs. 34.4% pandemic), buprenorphine (33.3% vs. 21.9%), gabapentin (38.9% vs. 3.1%), and clonidine (11.1% vs. 0%) used in a way other than how they were prescribed (Table A2). 

Results from the survey and qualitative interviews were grouped into the following themes: changing drug costs and formulations; increasing drug use and injection behaviors; perceived risk of overdose; use of harm reduction measures. Each of these is described below in a mixed-methods fashion. Pseudonyms were used in presenting the qualitative data.

### 3.2. Changing Cost and Formulation of Drugs

When queried on the impact of the pandemic on their drug use, 50% of respondents agreed or strongly agreed with the statement: *“The types of drugs I use has changed during this time due to availability,”* and 66% agreed or strongly agreed that *“The process of getting drugs has been more difficult during this time [between August 2020 and May 2021]”* (Table 2). Further qualitative exploration identified product cost as a driver of these changes. Individuals who predominantly used methamphetamines reported that the price of the drug had increased substantially during the pandemic. Owen stated *“It [methamphetamines] was a lot cheaper [before COVID-19]. Like you could get an eightball for $100. Then, corona hit and now it’s dollar for dollar [meaning a 3-fold increase in price].”* Michael, who reported that he began cooking methamphetamine again because he could not afford the higher prices, stated, *“I will say, this ice [slang for methamphetamine], like I stopped buying it actually, because it skyrocketed in price…overnight it went from $60 to $80, then to $200, like overnight…but, I know how to make my own.”*

Participants who predominantly used opioids noted that heroin had become scarce. In contrast, fentanyl beans or buttons, a formulation in which fentanyl powder is encapsulated in a gel coating, had flooded the market. As these fentanyl beans or buttons were relatively inexpensive and available, many participants reported a shift in their drug use away from heroin, or drugs sold as heroin, to fentanyl. Linda, who recently transitioned to fentanyl use, stated, *“Heroin is harder to find anymore. Over the summer…everybody went to [large local city] to get these beans, little capsules full of fentanyl. And they’re cheap, they’re $10, cheaper sometimes. And usually, that one little bean will get you high all day…compared to like $20 to $30 for heroin.”* Mason reported that fentanyl *“blew up everywhere”* following the onset of the pandemic. When asked about his experiences with fentanyl, Charlie stated, *“I started using fentanyl during COVID, so that’s all I know about it.”*

### 3.3. Increasing Drug Use and Injection Practices

Beyond changes in types of drugs used, many respondents reported increases in the frequency of their drug use. Participants indicated that time at home due to job loss or stay-at-home orders resulted in more free time and left them feeling bored. According to several participants, the excess downtime translated to increases in drug use. When asked if drug use had changed during COVID, Linda said, *“It seems like it’s increased, I think because everyone is inside more, and they get bored.”* Justin, a 33-year-old man stated, *“Yeah, it’s increased a little bit, I have more time on my hands”.*

Others noted that COVID-19 related stress had led to an increase in their drug use. Mason, who endorses fentanyl use, stated, *“Maybe it might have got worse…I probably do more drugs now.”* When asked about this change in drug use, the participant reported that stress likely contributed. Worsening economic status as a result of job loss was endorsed by many participants already vulnerable to financial and housing insecurity, with 62% of participants reporting homelessness in the past 6 months prior to their baseline assessment, and more than half disagreed with the statement that people in their community would have somewhere to shelter in place during lockdown (Table 2). These stressors amplified feelings of depression during the pandemic. Survey data illustrated these reports as 54% agreed or strongly agreed with the statement: *“I feel more depressed, unmotivated, or defeated during this time than I normally do”* (Table 2). Furthermore, 76% of survey respondents agreed or strongly agreed with the statement: *“I feel more anxious or on edge during this time than I normally do”* (Table 2). 

Some participants described changes in the route of administration as a result of the pandemic. For example, when asked what prompted a transition to injection drug use, Eva, a 30-year-old woman, stated, *“It’s just being cooped up in the house all the time, no job…I guess depression, too.”* In some cases, changes in drug availability and the increased cost of methamphetamine led to changes in administration methods, including injecting to optimize drug effects. Owen, who noted the higher price of methamphetamine, stated, *“Before the corona, I didn’t really know anybody around here that shot it [methamphetamine] up. Now everybody’s doing it that way.”*

### 3.4. Increasing Risk of Overdose 

Survey findings revealed both increasing experience with overdose and near overdose due to fentanyl exposure, as well as behavioral adaptations as a result of the perceived increase in risk. Overall, 50% of survey respondents were more likely to use alone since the pandemic, and over half were concerned about ending up *“with a bad batch of drugs that is dangerous”* (Table 2). Carla, who reported predominantly methamphetamine use, stated, *“…before the COVID, I never had it [fentanyl] as far as I know. And if I did, then I must not have had a high enough dosage because the last two times I’ve had it, it’s almost killed me. So more people [dealers] are starting to lace their stuff with fentanyl to try and up the quality of it to get more money out of it. And it’s, they’re killing people by doing that. So now I use [fentanyl] test strips on everything I get.”*

The influx of fentanyl beans during the COVID-19 pandemic led at least one participant to a safer route of drug administration. Charlie, who primarily used heroin and methamphetamine by injection before the pandemic began reported increased fentanyl overdose among peers. Charlie stated, *“I started doing like that [injecting], but they [fentanyl beans] were just shooting too strong, people were falling out on them, so I just started snorting them.”*

### 3.5. Access to Harm Reduction Services

While many harm reduction programs across the US scaled back services or experienced closures following the onset of the pandemic, the referring organization for ETHIC, the Community Action Place, Inc., (tCAP), remained operational and increased hours and their geographic service area. tCAP is a nonprofit, community-based organization that provides harm reduction services such as sterile syringes and equipment, pipes and pipe covers, cotton, fentanyl test strips, naloxone, sexual safety supplies such as condoms, lubricants, and dental dams, and testing for infectious diseases. Services are free of charge and confidential. Serving the southernmost 19 counties in Illinois, tCAP operates from a mobile model in which staff delivers services to clients in their homes and communities, working to accommodate transportation-related barriers to service uptake. During the pandemic, tCAP modified its service delivery protocol to meet COVID-19 safety guidelines, implementing the use of personal protection equipment (PPE), social distancing, and contactless delivery. 

Survey data indicated that 96% of participants were confident they could obtain sterile syringes and injection equipment during the COVID-19 pandemic, and 90% were confident they could obtain naloxone during this time (Table 2). Although 76% of participants reported engaging in any injection equipment sharing practices (Table 1), this was largely related to the sharing of cotton, cookers, and water. There was limited use of shared syringes or needles among participants, indicating that perceptions of risk behaviors may be hierarchical, i.e., sharing preparation equipment may be seen as less risky, compared with sharing syringes and needles. Furthermore, sharing preparation equipment may be expected in communal use settings where the quantity of drugs is limited. 

Additionally, 96% of participants agreed or strongly agreed that they could “*rely on tCAP as a good source of information about COVID-19”* (Table 2). Of those who completed the COVID-related survey and/or interview, 40% were not previously clients of the harm reduction organization. All of these participants accepted referral to the organization during COVID-19, after engagement with the ETHIC study. 

The importance of accessible harm reduction services was reflected in a number of interviews. Shaun, a new client of tCAP who reported having shared and reused injection equipment prior to becoming a client said, *“There’s been a lot less concern about where all these people are going to get their needles because there are a lot more of them out there. New ones, which is amazing. Um, this Narcan [naloxone] is, uh, so much easier to get ahold of, which has been amazing. Um, I’ve used it many a time. Yeah, they’re [tCAP staff] lifesavers.”* When asked what she liked most about tCAP, Kim, a more established client, said, *“I always say the onsite [HIV/HCV/STI] testing and supplies.”* Additionally, fentanyl test strips were widely discussed by participants as important resources, giving them more ability to adapt their drug use practices based on results. Caroline, who endorsed methamphetamine injection and had recently been linked to services, stated, *“Here recently, there may have been a time or two that, uh, it [methamphetamine] was laced [with fentanyl]. Something I got, and basically, it just put me to sleep…Uh, and I didn’t actually know that they [dealers] were lacing with fentanyl until I talked to [tCAP staff] and he told me and basically told me how to use it….I definitely want to be informed on what I’m doing.”* Not only did participants vocalize the importance of formal harm reduction services offered by the local organization, Caroline, as well as others, reported relying on staff as a source of informal information about potential harms of drugs in the area. 

Furthermore, many participants indicated that accessing the harm reduction organization provided them with social support that was often otherwise unavailable. Charlie, another new client, stated, *“This [COVID-19] is kinda wearing me down, and, um, you know, just with everything adding up. So, starting tCAP just, um, is kind of like a beacon of light for me. Like I mean, it makes you feel like there is hope, you know?”* Charlie went on to discuss the importance of the organization for his rural community at large, saying *“[name of county] is one of the poorest counties in the state, and possibly in the nation, in my opinion, and we need all the help we can get. So, I think it’s a wonderful thing…you know, like the majority of people don’t give a fuck about drug users…and I think it’s awesome that somebody’s looking out for the little guy…I’m actually proud of myself being a part of the program.”* Lisa, a relatively new client of the organization, noted that she felt that drug-use stigma was reduced by tCAP’s work, said, *“I’m excited today…This tCAP is something that when I moved here, I couldn’t believe wasn’t available…the ones that really needed it [harm reduction services] weren’t aware, and therefore, we still have a problem, and it continued to grow. I feel like the way that things are being done that, uh, a lot of the stigma has been removed. I’m excited for the program to be here.”*

## 4. Discussion

Our exploratory examination of drug use changes after the onset of the COVID-19 pandemic among a cohort of people living in rural Illinois who use drugs found increasing use of fentanyl, both intentional and unintentional, with a corresponding perceived increased risk of overdose. This echoes state-level data showing increasing rates of opioid-related overdose during the pandemic and mirrors national trends of increasing fatal overdose being driven by synthetic opioids including fentanyl, as well as increasing stimulant use. Participants identified an increasing cost and decreasing availability of heroin and methamphetamine, concerns about fentanyl contamination of methamphetamine, and an influx of less expensive fentanyl products, including gel-encapsulated “beans” and “buttons”, as factors leading to more fentanyl exposure during the COVID-19 pandemic. It is worth noting that we are unaware of other reports in the research literature that have described fentanyl “beans” and “buttons” being marketed as fentanyl—whether these are slang terms for more common pill formulations of the drug or truly formulations more unique to this geographic setting warrants further investigation. Rising drug prices reported here may shift substance use to less-familiar products and/or sources and promote potential riskier modes of production (of methamphetamines), as well as generate additional financial pressure during a time of increasing economic instability [23,24]. Additionally, participants reported less nonmedical use of prescribed medications (benzodiazepines, buprenorphine, gabapentin, etc.) during the pandemic. Whether this could be related to reduced access to medical care, concerns about seeking care during the pandemic, or other reasons, merits further study.

Outside of shifting drug markets, study participants described concerning changes in drug use behavior that may exacerbate the risk of overdose. As has been previously reported [25,26], increasing stress, anxiety, and lack of employment, coupled with the social isolation brought on by COVID-19 risk, stay-at-home orders, and subsequent job loss, led to increased frequency of use, as well as more frequent use alone. Data from Italy, for example, showed a shift away from less-available drugs to other types of drugs, as well as increased use even after their lockdown ended [27]. These findings highlight the critical need to increase social support, mental health care, and access to overdose prevention, including naloxone for this vulnerable population during and beyond the pandemic. 

Notably, some participants described the adoption of protective adaptations in their drug use, including the use of fentanyl strips to test products as well as modifications in the method of administration (insufflation vs. injection), to mitigate the risk of overdose. Sterile syringes, naloxone, and infectious disease testing were also important to clients. Perhaps more salient, participants described their engagement in harm reduction services as a source of pride, validation, and dignity, and some noted that the organization’s resiliency during the pandemic provided participants with a sense of hope. These findings expand on previous studies highlighting the importance of syringe service programs [28,29,30]. Our findings are consistent with other rural settings in which the ability of syringe service programs to pivot and adapt quickly during the COVID-19 pandemic has been a great advantage to the community [25,31]. Research on how programs may leverage these strengths to expand scopes of service in the pandemic era should be prioritized. For example, the success of telemedicine in providing opioid use disorder treatment in rural areas [32], in concert with evolving policies related to reimbursement, provides an opportunity to explore how novel mobile and other low-threshold care may be linked to or delivered through harm reduction programs. Given high rates of governmental distrust in people who use drugs, particularly those in rural communities, harm reduction service providers as trusted messengers could be empowered to provide public health messaging around COVID-19 information and vaccination [33,34].

## 5. Conclusions

Our findings indicate that exposure to fentanyl through gel-encapsulated “beans” or “buttons” increased during the pandemic period as heroin became more difficult to procure and methamphetamine costs increased. Further, participants reported being more likely to use drugs alone than prior to the pandemic and increased witness and/or experience of overdose. Additionally, participants vocalized increased feelings of anxiety, loneliness, and depression since the onset of the COVID-19 pandemic. Uninterrupted access to harm reduction services, delivered through a mobile model, during the pandemic provided participants with life-saving preventive measures such as fentanyl test strips for drug checking and naloxone to reverse an opioid-related overdose. Additionally, participants expressed relying on the local harm reduction agency as a trusted source of information about COVID-19 and accompanying safety guidelines (e.g., masking, social distancing). 

## Figures and Tables

**Table 1:** Participant baseline characteristics (*n* = 50), ETHIC study, southern Illinois 2018–2021.

	*n* (%)
**Age** (mean, SD)	40.4 (SD 10.0)
**Gender**	
Male	26 (52.0)
Female	24 (48.0)
**Race**	
White	44 (88.0)
Black	5 (10.0)
American Indian	1 (2.0)
**Education**	
Elementary	7 (14.0)
Some high school	21 (42.0)
High school graduate	16 (32.0)
Some college/technical	5 (10.0)
Missing	1 (2.0
**Homelessness in past 6 months**	31 (62.0)
**Accessed food supports in past 6 months**	46 (92.0)
**Size of social network (mean, SD)**	12.2 (SD 9.2)
**Drug Use**	
Injected drug use	50 (100.0)
Inject more than one time in a single sitting in the last 30 days, from the same solution	27 (58.7)
**Participant-reported drug use in the last 30 days** *	
Heroin	26 (60.5)
Fentanyl	19 (45.2)
Opiate pain killers ^††^	18 (37.5)
Synthetics ^‡^	2 (6.0)
Buprenorphine ^§^	13 (29.6)
Methadone ^§^	1 (2.5)
Prescription anxiety drugs ^†††^	18 (39.1)
Cocaine or Crack	15 (30.6)
Methamphetamines	48 (96.0)
Gabapentin ^§^	8 (18.2)
Clonidine ^§^	2 (5.4)
**Sharing Practices in the last 30 days**	
Use a syringe/needle that you know used by someone else	8 (17.4)
Use a cotton/cooker/spoon rinse by somebody else	18 (39.1)
Let someone else use a cotton/cooker/spoon after using it	14 (30.4)
Inject drugs that somebody else prepared, mixed, or divided with a used syringe	9 (19.6)
Any of the above	35 (76.1)
**Overdose**	
Ever experienced an overdose	27 (77.1)
Lifetime number of overdose(s) experienced (mean, SD)	3.5 (SD 6.1)
Witnessed an overdose	38 (76.0)
**Source of needles/syringes**	
Pharmacy	29 (63.0)
Syringe or needle exchange program	29 (58.0)

* Last 30 days use from the time of baseline survey; ^††^ i.e., oxycodone, Percocet, hydroxycodone, Vicodin, Lortab, etc. used in a nonprescribed manner; ^†††^ i.e., Xanax, Valium, Klonopin, used in a nonprescribed manner; ^‡^ i.e., U47700, U4, or “Pink”; ^§^ Used in a nonprescribed manner.

**Table 2:** COVID-19 survey responses (*n* = 50).

	Agree		Neutral		Disagree		Do Not Know	
Question	*n*	%	*n*	%	*n*	%	*n*	%
**Drug Use and Drug Use Community**								
Most people who use drugs have somewhere they can shelter in place during this time.	17	34.0	3	6.0	28	56.0	2	4.0
When I get drugs, I have not been able to follow social distancing recommendations (e.g., staying six feet or more away from others, avoiding crowded places).	24	48.0	3	6.0	22	44.0	1	2.0
The type of drugs I use has changed during this time due to availability.	25	50.0	2	4.0	22	44.0	1	2.0
The process of getting drugs has been more difficult during this time	33	66.0	2	4.0	14	28.0	1	2.0
Because of less than normal supply, I feel pressure to share drugs, supplies, and equipment.	15	30.0	2	4.0	32	64.0	1	2.0
I am more likely to use drugs alone during this time than I was before.	25	50.0	3	6.0	21	42.0	1	2.0
I worry that I will end up with a bad batch of drugs that is dangerous in the near future.	28	56.0	10	20.0	11	22.0	1	2.0
**Mental Health**								
I feel more depressed, unmotivated, or defeated during this time than I normally do.	27	54.0	2	4.0	21	42.0	0	0.0
I feel lonelier during this time than I normally do.	20	40.0	4	8.0	26	52.0	0	0.0
I feel more anxious or on edge during this time than I normally do.	38	76.0	2	4.0	10	20.0	0	0.0
**Harm Reduction Access**								
I can rely on tCAP as a good source of information about COVID-19.	48	96.0	1	2.0	0	0.0	1	2.0
I’m confident I can obtain naloxone/Narcan during this time.	45	90.0	1	2.0	3	6.0	1	2.0
I’m confident I can obtain sterile syringes and injection equipment during this time.	48	96.0	0	0.0	1	2.0	1	2.0
I’m confident I can access Scott and tCAP and their services/resources during this time.	48	96.0	0	0.0	1	2.0	1	2.0

## Appendix A

**Table A1:** Timing of participant completion of baseline assessment: COVID-19 survey and qualitative interviews.

	Pre-Pandemic Period	Pandemic Period
	June 2018–May 2019	August 2020–May 2021
Baseline Survey (*n* = 50)	18	32
COVID-19 Survey (*n* = 50)		50
Qualitative Interviews (*n* = 19) *		19

* Five qualitative interviews did not complete the COVID-19 survey.

**Table A2:** Participant baseline characteristics by assessment period, pre-pandemic, and pandemic period.

	Pre-Pandemic (*n* = 18)	Pandemic (*n* = 32)
**Age** (mean, SD)	41.4 (SD 11.1)	39.8 (SD 9.4)
**Gender**		
Male	9 (50.0%)	17 (53.1%)
Female	9 (50.0%)	15 (46.9%)
**Race**		
White	17 (94.4%)	27 (84.4%)
Black	1 (5.6%)	4 (12.5%)
American Indian	0 (0.0%)	1 (3.1%)
**Education**		
Elementary	1 (5.6%)	6 (18.8%)
Some high school	8 (44.4%)	13 (40.6%)
High school graduate	6 (33.3%)	10 (31.3%)
Some college/technical	3 (16.7%)	2 (6.3%)
Missing	0 (0.0%)	1 (3.1%)
**Homelessness in past 6 months**	12 (66.7%)	19 (59.4%)
**Accessed food supports in past 6 months**	16 (88.9%)	30 (93.8%)
**Size of social network (mean, SD)**	12.0 (SD 9.5)	12.4 (SD 9.2)
**Drug Use**		
Injected drug use	16 (88.9%)	32 (100.0%)
Inject more than one time in a single sitting in the last 30 days, from the same solution	8 (57.1%)	19 (59.4%)
**Participant-reported drug use in the last 30 days** ^†^		
Methamphetamines	17 (94.4%)	31 (96.6%)
Heroin	8 (72.7%)	18 (56.3%)
Prescription anxiety drugs ^††^ *	7 (50.0%)	11 (34.4%)
Opiate pain killers ^†††^	10 (62.5%)	8 (25.0%)
Buprenorphine ^§^ *	6 (33.3%)	7 (21.9%)
Gabapentin ^§^ **	7 (38.9%)	1 (3.1%)
Cocaine or Crack	7 (41.2%)	8 (25.0%)
Fentanyl	4 (40.0%)	15 (46.9%)
Clonidine ^§^ *	2 (11.1%)	0 (0.0%)
Methadone ^§^	1 (5.6%)	0 (0.0%)
Synthetics ^‡^	1 (5.6%)	1 (3.1%)
**Sharing Practices in the last 30 days**		
Use a syringe/needle that you know used by someone else	3 (21.4%)	5 (15.6%)
Use a cotton/cooker/spoon rinse by somebody else	6 (42.9%)	12 (37.5%)
Let someone else use a cotton/cooker/spoon after using it	5 (35.7%)	9 (28.1%)
Inject drugs that somebody else prepared, mixed, or divided with a used syringe	3 (21.4%)	6 (18.9%)
Any of the above	12 (85.7%)	23 (71.9%)
**Overdose**		
Ever experienced an overdose **	9 (52.9%)	18 (56.2%)
Lifetime number of overdose(s) experienced (mean, SD)	3.0 (SD 3.4)	3.8 (SD 6.9)
Witnessed an overdose	12 (66.7%)	26 (81.3%)
**Source of needles/syringes**		
Pharmacy	10 (71.4%)	19 (59.4%)
Syringe or needle exchange program	9 (50.0%)	20 (62.5%)

* *p* < 0.05 and ** *p* ≤ 0.01; ^†^ Last 30 days use from the time of baseline survey; ^††^ i.e., Xanax, Valium, Klonopin, used in a nonprescribed manner; ^†††^ i.e., Oxycodone, Percocet, Hydroxycodone, Vicodin, Lortab, etc. used in a nonprescribed manner; ^‡^ i.e., U47700, U4, or “Pink”; ^§^ Used in a nonprescribed manner.
